# The PoV mycovirus affects extracellular enzyme expression and fruiting body yield in the oyster mushroom, *Pleurotus ostreatus*

**DOI:** 10.1038/s41598-020-58016-4

**Published:** 2020-01-23

**Authors:** Ha-Yeon Song, Nayeon Kim, Dae-Hyuk Kim, Jung-Mi Kim

**Affiliations:** 1 Department of Bio-Environmental Chemistry, Institute of Life Science and Natural Resources, Wonkwang University, Iksan, 54538 Korea; 2 Department of Molecular Biology, Department of Bioactive Material Sciences, Institute for Molecular Biology and Genetics, Chonbuk National University, Jeonju, Chonbuk 54896 Korea

**Keywords:** Applied microbiology, Fungal biology, Viral host response

## Abstract

Isogenic virus-cured and virus-infected fungal strains were previously obtained and compared to investigate mycoviral diseases and, specifically, the influence of viral infection on the vegetative growth of *Pleurotus ostreatus*. The present study demonstrated that infection with mycovirus PoV-ASI2792 (PoV) caused phenotypic and physiological changes in fungal cells and mycelia. The microscopically determined growth rate of the virus-infected strain was lower than that of the virus-cured strain, due to the conglomerate phenomenon during the mycelial growth process. An exploration of the viral effects of PoV on fruiting bodies yield showed significantly lower than that on virus-cured *P. ostreatus*. A colorimetric assay of polyphenol oxidase activity in the strains showed very weak activity in the virus-infected strain. To estimate the activity levels of enzymes related to the growth and fruiting body formation, the relative expression levels of genes encoding various extracellular enzymes such as Carbohydrate-Active Enzymes (CAZymes**)** were measured by quantitative RT-PCR. The expression levels of the assayed genes were significantly lower in virus-infected than in virus-cured *P. ostreatus*. Together, these results indicate that PoV infection affects the spawn growth and fruiting body formation of *P. ostreatus via* decreased expression and activity of some extracellular enzymes including lignocellulolytic enzymes.

## Introduction

Mycoviruses infect and replicate in all major fungal taxa, including both pathogenic and edible fungi. The fungal viruses are commonly latent, and hosts do not exhibit symptoms upon infection. Nevertheless, some mycoviruses cause severe morphological and physiological changes in fungal hosts, such as alterations of growth rate, colony morphology, spore production, pigmentation^1–3^, and enzymatic activities^4^. The first reported mycovirus (La France isometric virus, LIV) was found in 1962 in the cultivated mushroom *Agaricus bisporus*. The mushroom virus causes abnormal mycelial growth and malformed fruiting body production^5^.

In the edible mushroom *Pleurotus ostreatus*, mycoviral infections can cause symptoms such as irregular mycelial growth rate, decreased fruiting body yield, and malformed fruiting bodies, and thus occasionally affect cultivation of the mushroom. As mentioned above, several mushroom diseases have been reported to be associated with mycoviruses, such as oyster mushroom spherical virus (OMSV)^6^, oyster mushroom isomeric virus (OMIV)^7^, *P. ostreatus* spherical virus (POSV)^8^, oyster mushroom dsRNA virus (OMDV)^9^, *P. ostreatus* virus 1 (PoV1)^10^, and *P. ostreatus* ASI2792 virus (PoV-ASI2792; PoV)^3^.

Mycoviruses infecting various hosts including *P. ostreatus* have been isolated and characterized. However, studies of mycovirus–host interactions have been insufficient to reveal the mechanisms involved in infection of *P. ostreatus* because it is difficult to secure definite isogenic virus-free or -cured strains to compare to virus-infected strains^3^. Because different genotypes of the same fungal species can produce inconsistent data with regard to identical mycovirus strains, various approaches have been used to obtain isogenic virus-cured strains, such as cycloheximide or ribavirin treatment, hyphal tip cutting, single-sporing, protoplast regeneration, or thermal stress treatment^11–14^. Even when the virus is successfully cured from a virus-infected fungal cell, impairments caused by the treatment make the cured fungal cell inappropriate for investigations of fungal–mycoviral interactions. In the method for securing an artificial virus-infected strain, infectious cDNA clones of a fungal virus for artificial infection were transformed into the virus-cured fungal strain upon integration of the infectious clones into the fungal genome based on Koch’s postulates^15^. However, this method is also challenging because of the difficulty in the case of multi-segmented mycovirus^15^ and the alteration of gene expression with non-homologous chromosomal integration, such as ectopic recombination^16^. Therefore, in our previous investigation of PoV in *P. ostreatus*, virus-curing was performed by the mycelial fragmentation method followed by single colony isolation^17^. A virus-cured strain (free version) of a naturally infected isolate can be obtained by a technique based on selecting progeny or fungal hyphae without the virus^15^. Virus-cured isogenic lines, which have identical genetic backgrounds, can then be established to explore direct mycovirus–fungal host interctions. As reported, we successfully obtained a PoV-cured strain as well as the PoV-infected strain from a malformed fruiting body, and verified the presence of the virus and its effect on the irregular colony growth of *P. ostreatus*^3^.

*Pleurotus ostreatus*, a white-rot basidiomycete, degrades its substrates (sawdust, beet pulp, rice bran, rice straw, cotton-seed, etc.) with various carbohydrate-active enzymes (CAZymes) such as lignocellulolytic enzymes associated with lignin degradation^18^. CAZymes secreted by fungi are classified into several families, including two high-priority classes, i.e., glycoside hydrolase (GH) and auxiliary activities (AA)^18^. These enzymes assist in the acquisition of nutrients to enhance mycelial growth and fruiting body production in *Pleurotus* spp. The expression levels of genes encoding extracellular enzymes associated with the degradation of lignin, cellulose, hemicellulose, and pectin exhibit clear distinctions depending on the culture conditions, such as available nutrients (substrate composition); temperature; levels of moisture, light, carbon, and oxygen; and presence of infectious diseases, in cultivated mushrooms^19,20^. Because it is difficult to prevent disease in mycovirus-infected strains of cultivated mushrooms, other fungal species have been used to investigate the gene expression and activity levels of extracellular enzymes, such as lignocellulolytic enzymes, in mycovirus-infected strains^4^. However, there is little literature about the effect of mycoviruses on the gene expression levels and activities of extracellular enzymes, even though mycoviruses can cause drastically decreased fruiting body production in cultivated mushrooms, including *Pleurotus* spp.

In the present study, we explored the novel biological functions of PoV, which causes phenotypic aberrations or alterations in fungal growth and fruiting bodies, through the comparison of isogenic virus-cured and -infected strains of *P. ostreatus*. Expression and activity levels of extracellular enzymes such as lignocellulolytic enzymes were analyzed throughout PoV infection of *P. ostreatus* to investigate the relationship between the mycovirus and the discernible phenotypic changes in growth and fruiting bodies. To our knowledge, this is the first report describing the effects of a mycovirus infection on the gene expression of representative CAZymes (CAZy GH and CAZy AA class enzymes) and non-CAZymes during the different developmental stages of *P. ostreatus*.

## Results

### Morphological abnormality of the PoV-infected strain

In our initial survey of dysmorphic fruiting bodies of *P. ostreatus* ASI2792, we obtained abnormal mycelial cells, confirmed the presence of the PoV mycovirus (PoV-ASI2792)^3^, and examined the deleterious effects of mycoviral infection on the growth of fungal colonies using isogenic virus-cured and virus-infected fungal strains. In the present study, we attempted to better understand the relationship between PoV infection and the vegetative characteristics of *P. ostreatus* based on a pair-wise comparison of isogenic strains. Two virus-cured lines, *P. ostreatus* AS2792-VF10 (POVF10) and *P. ostreatus* AS2792-VF12 (POVF12), were randomly selected on the basis of normal phenotypes using previous data^3^. Whenever a comparative analysis of isogenic strains is performed, the status of PoV infection should be confirmed based on colony morphology and viral genome assay. For this comparative study of one PoV-infected line (POV) and the two virus-cured lines (POVF10 and POVF12), the presence or absence of the PoV mycovirus was verified for each strain *via* gel electrophoresis and RT-PCR using *P. ostreatus* mycelium. The genome segment and a partial cDNA from the PoV mycovirus were only observed in the mycelia from the virus-infected strain (Supplementary Fig. S1).

**Figure 1:**
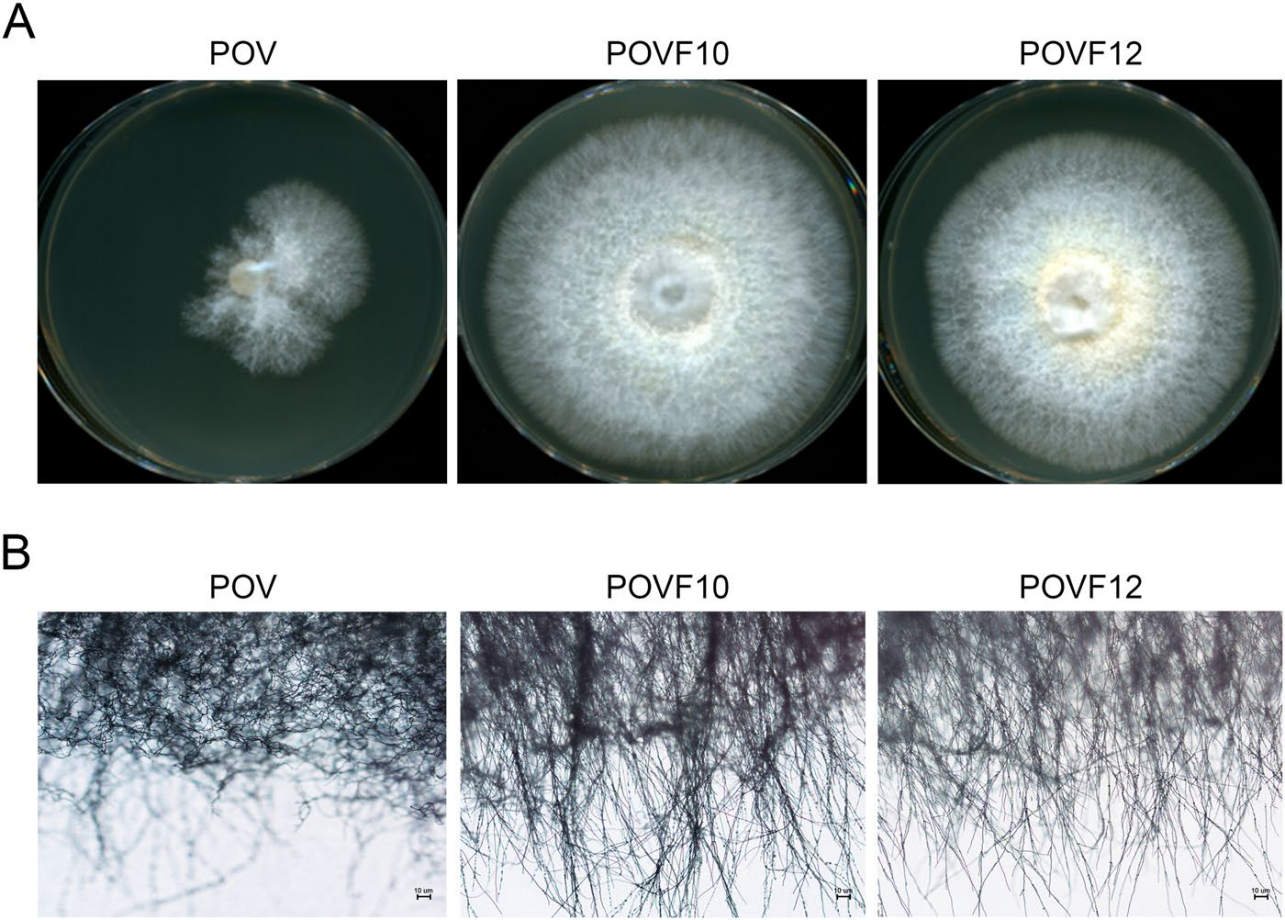
Characteristics of mycelial growth in a mycovirus-infected *P. ostreatus* strain. (**A**) Colony morphology of the PoV-infected strain was compared to that of wild-type *P. ostreatus* (virus-cured strain) after 7 days of incubation at 25 °C. The strains, as indicated on the panels, are a PoV-infected strain (POV) and two virus-cured strains (POVF10 and POVF12)^3^. (**B**) Microscopic features of the colony margin in the PoV-infected strain were compared to those of virus-cured strains after 2 days of incubation at 25 °C. Black bars = 10 μm (200× magnification).

The POV strain, which contained the PoV mycovirus and exhibited reduced mycelial growth, was compared to the two virus-cured strains, POVF10 and POVF12, using microscopic observation (Fig. 1). As previously reported, the colony morphology of the POV strain differed from that of the virus-cured strains. Colony growth of the POV strain decreased significantly, resulting in irregular margins (Fig. 1A). Because the colony margin appeared unusual, we investigated the branching pattern of marginal hyphae. Both virus-cured strains, POVF10 and POVF12, exhibited a regular branching pattern, whereas the POV strain developed a denser network of fungal hyphae due to a hypertwisting pattern (Fig. 1B). The mycelial growth rate of the POV strain was markedly lower than those of the POVF10 and POVF12 strains due to its short and agglomerative hyphal branching.

To examine the effect of viral infection on cell wall integrity, the virus-infected and -cured strains were cultured on media containing the cell wall-disturbing agents. No considerable change was observed in response to either agent (Supplementary Fig. S2), suggesting that PoV infection is unrelated to cell wall integrity in the POV strain. The mycelial growth rate of the POV strain decreased by ~40% when it was grown on sawdust agar plates under the same conditions as in PDA culture. In addition, different concentrations of sawdust (5, 10, 15, 20, and 25%) had no effect on the mycelial growth rate of the POV strain (Supplementary Fig. S3).

### PoV infection reduced fruiting body yield

The mushroom weight of *P. ostreatus* depends on the morphology and number of effective fruiting bodies per sawdust bottle. Mushroom weight is closely related to yield and biological efficiency. To investigate the effect of PoV infection on fruiting body production and yield, the fruiting bodies of the virus-infected strain (POV), which exhibited delayed mycelial growth caused by abnormal hyphal branching, were compared to those of two virus-cured isogenic strains, POVF10 and POVF12. The highest yield of all tested mushroom strains was obtained from the first flush, and yields gradually reduced upon subsequent flushes. Fruiting bodies of the POV strain were only obtained from three flushes, whereas fruiting bodies of the POVF10 and POVF12 strains were obtained from more than four flushes (Tables 1 and S2). The reduction in flush efficiency (FE) began early in the POV strain, starting with the second flush stage. The FE ratio of the POV strain was reduced by ≥66.7% by the third flush, in comparison to the two virus-cured strains. Thus, PoV infection increased the reduction rate in FE in *P. ostreatus* (Supplementary Table S2).

**Table 1:** Effects of PoV infection on mushroom yield and biological efficiency in *P. ostreatus*.

Strains	Fresh weight of mushroom by flushed (g/bag)				
	1st flush	2nd flush	3rd flush	4th flush	Total yield
POV	93.51^a^	26.85^a^	3.31^a^	0.00^a^	123.67^a^
POVF10	98.93^a^	61.25^b^	15.04^ab^	4.09^a^	180.80^b^
POVF12	98.30^a^	60.25^b^	23.09^b^	3.10^a^	185.88^b^

Different superscript letters indicate significantly different means within the same column (p < 0.05).

**Figure 2:**
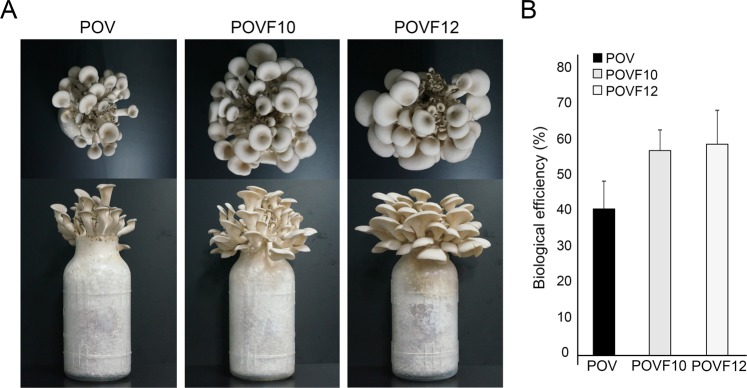
The effects of the PoV mycovirus on fruiting body formation in *P. ostreatus*. (**A**) The morphological characteristics of fruiting bodies of a virus-infected strain (POV) and two virus-cured strains (POVF10 and POVF12) cultivated on sawdust were observed. These were fruiting bodies from the second flush stage. (**B**) The fruiting body yield of each *P. ostreatus* strain was measured to determine the biological efficiency (BE; %) of fruiting bodies from the POV, POVF10, and POVF12 strains. BE was calculated as the ratio of fresh weight of harvested mushrooms to substrate dry weight. BE was compared between strains using Duncan’s multiple range test. A value of p < 0.05 denotes statistical significance. The black bar indicates analyses of the POV strain. The other two bars indicate analyses of the virus-cured POVF10 and POVF12 strains.

Over time, fruiting bodies of the POV strain exhibited symptoms such as short and plump stems, and few and small caps, compared to the two virus-cured strains (Fig. 2A). For these reasons, mushroom weights between the virus-infected and -cured strains were significantly different after the first flush (Table 1). The mushroom weights from the second and third flushes of the POV strain were reduced to 55.4–56.2% and 78.0–85.7% of those of the virus-cured strains, respectively. The total fresh mushroom yield of the POV strain decreased by ≥31.6% compared to those of the POVF10 and POVF12 strains (Table 1). In the final analysis, the biological efficiency (BE) of the POVF10 and POVF12 strains ranged from 57.6% to 59.2%, and the BE of the POV strain was reduced by ≥32% compared to those of the two virus-cured strains (Fig. 2B). Overall, PoV infection affected the total yield and BE during the process of fruiting body production in *P. ostreatus*.

### Differences in viral titer between *P. ostreatus* mycelia and fruiting bodies

**Figure 3:**
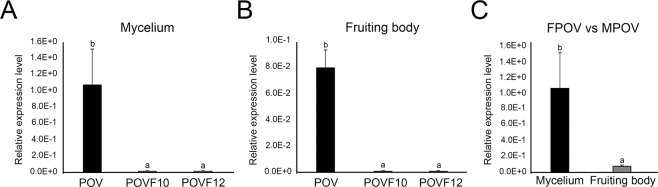
Relative transcript abundances of the PoV gene in *P. ostreatus* based on quantitative real-time polymerase chain reaction (qRT-PCR). Expression levels of the PoV gene in dsRNA extracts from mycelia (**A**) and fruiting bodies (**B**) of the virus-infected strain (POV) and the virus-cured strains (POVF10 and POVF12) were analyzed. In both assays, a housekeeping gene (actin) was used as an internal control. The black bar indicates analyses of the POV strain. The other two bars indicate analyses of the virus-cured POVF10 and POVF12 strains. To detect PoV, qRT-PCR was performed using three biological and technical replicates. (**C**) Results from qRT-PCR analysis of the PoV mycovirus in the mycelium (MPOV) and fruiting body (FPOV) of the POV strain. The actin gene was used as an internal control. The *y*-axis represents the expression levels of the genes relative to the actin gene in *P. ostreatus*. The black and light gray bars indicate analyses of the POV and POVF10 and POVF12 strains, respectively. A value of p < 0.05 denotes statistical significance.

To measure the relative accumulation of PoV RNA during two developmental stages of *P. ostreatus*, analysis of viral gene expression in the mycelium or fruiting body was performed using qRT-PCR. The results showed that the relative expression level of RdRp mRNA from PoV differed between the POV and POVF10/POVF12 strains. In general, there was no accumulation of PoV RNA in the mycelia or fruiting bodies of the virus-cured strains (POVF10 and POVF12), whereas PoV RNA accumulation in the POV strain was significant throughout two developmental stages of *P. ostreatus* (Fig. 3A,B). Interestingly, quantification data revealed that there was >13 times more RNA at the mycelial stage than at the fruiting body stage. The accumulation of PoV RNA at the fruiting body stage was remarkably lower than that at the mycelial stage (Fig. 3C). The PoV virus also exhibited significantly higher RNA levels in the virus-infected strain.

### Effect of PoV infection on phenol oxidase activity for lignin biodegradation

**Figure 4:**
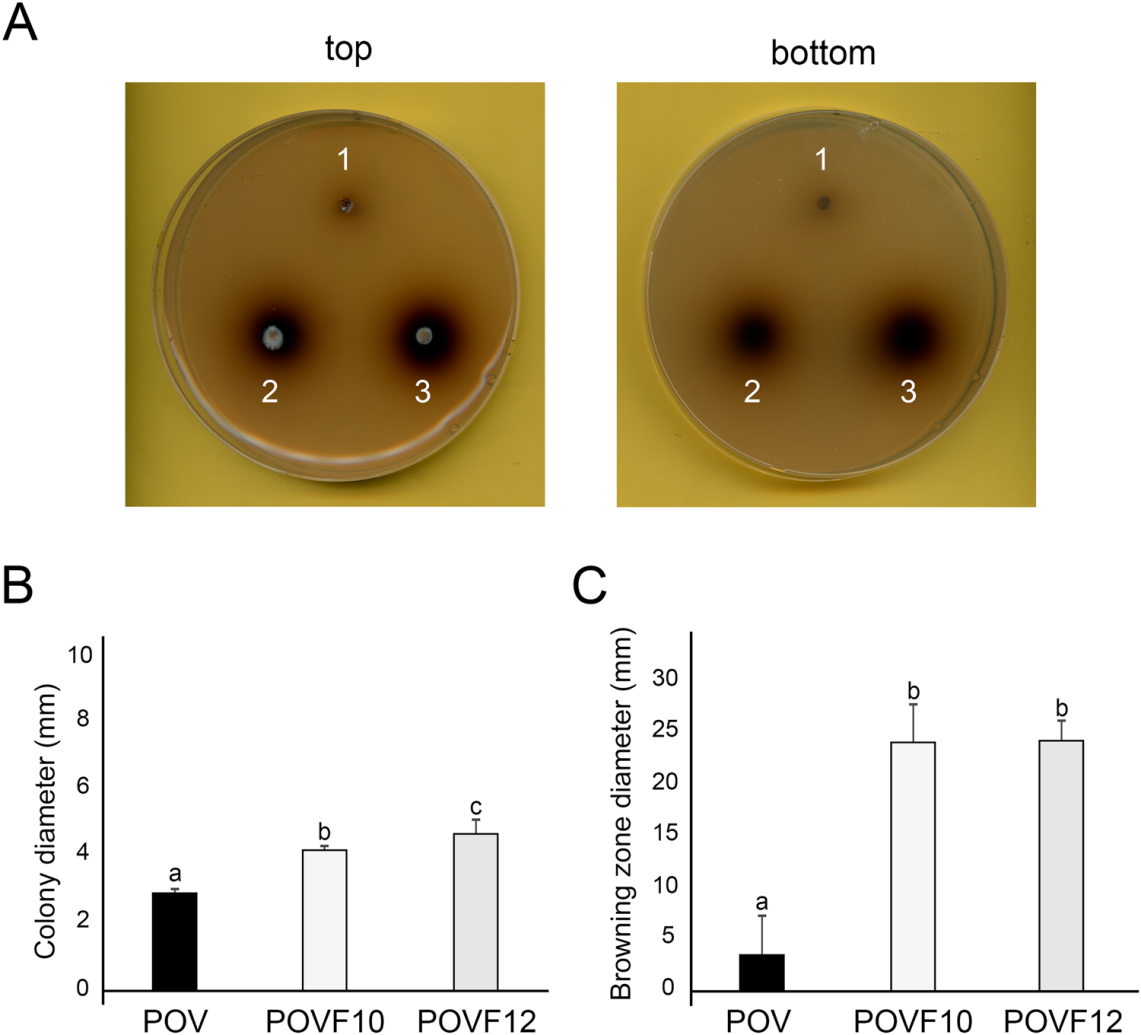
Bavendamm assay of a mycovirus-infected *P. ostreatus* strain. (**A**) Colonies were grown for 5 days on tannic acid-containing medium. The intensity of brown coloration correlates to the polyphenol oxidase activity in each strain. Top and bottom views of the Bavendamm’s plates are shown. The numbers 1, 2, and 3 indicate the virus-infected POV strain, and virus-cured POVF10 and POVF12 strains, respectively. (**B**) The diameters of the fungal colonies of the POV, POVF10, and POVF12 strains. The colony diameter is measured using the length of the agar block. (**C**) The diameters of the brown zones produced by the POV, POVF10, and POVF12 strains. The black bar indicates analyses of the POV strain. The other two bars indicate analyses of the virus-cured POVF10 and POVF12 strains. Data are presented as the means ± standard deviations from four replicates and three independent experiments.

To measure the effects of PoV infection on extracellular lignocellulolytic enzyme activities, the virus-infected and -cured strains were grown on Bavendamm’s medium. The POV strain produced an extremely small or no brown zone, and the size and color intensity of the zone were compared to those of the POVF10 and POVF12 strains (Fig. 4). The POV strain exhibited decreased growth rate and a smaller brown-colored zone under standard growth conditions. The intensity of the brown coloration produced by the POVF10 and POVF12 strains was far greater than that produced by the POV strain (Fig. 4A). The area of the dark brown zone, which is indicative of phenol oxidase secretion, produced by the POV strain was significantly smaller (by ≥86.5%) compared to those produced by the two virus-cured strains, POVF10 and POVF12 (Fig. 4C). Compared to the decreasing colonial growth rate, that of the brown zone declined significantly (Fig. 4B,C). PoV infection of *P. ostreatus* caused a large reduction or deficit in the size and color intensity of the brown zone created by phenol oxidase production.

### Effect of PoV infection on transcript accumulation of extracellular enzymes

**Figure 5:**
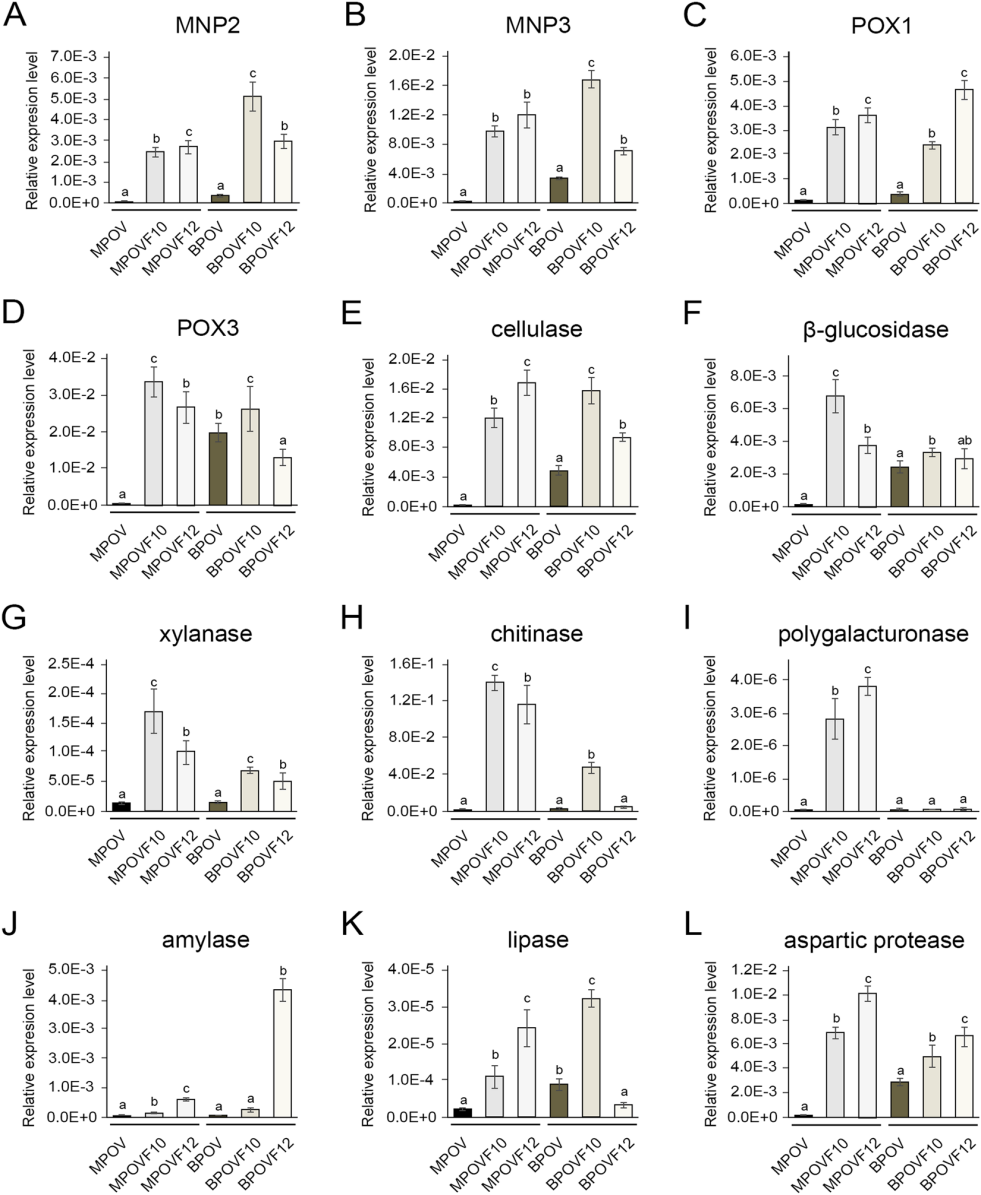
Relative expression levels of genes encoding extracellular enzymes during the growth stage in a mycovirus-infected *P. ostreatus* strain. The expression levels of 12 enzyme-encoding genes from the vegetative mycelium (M) and sawdust spawn (B) of the virus-infected strain (POV) were compared to those of the virus-cured strains (POVF10 and POVF12). Detailed primer information is listed in Supplementary Table S1. Each error bar represents the standard deviation calculated from at least three independent replicates. Transcript abundances of the 12 genes relative to a housekeeping gene (*cyt-c*) were quantified using qRT-PCR. The black and brown bars indicate analyses of the vegetative mycelium (MPOV) and sawdust spawn (BPOV) in the POV strain, respectively.

To investigate the effects of PoV infection on the activities of CAZymes, which are involved in lignin, cellulose, hemicellulose, pectin, and chitin degradation, as well as non-CAZymes associated with mycelial growth and fruiting body production, the relative expression levels of genes encoding extracellular enzymes in the virus-cured (POVF10 and POVF12) and -infected (POV) strains were analyzed using qRT-PCR. Total RNA from three developmental stages—vegetative mycelium (M), mycelium spawn (B), and mature fruiting body (F)—was used to compare the mRNA expression of 12 genes encoding 10 different extracellular enzymes (amylase, aspartic protease, cellulase, chitinase, β-glucosidase, laccases, lipase, manganese peroxidases, polygalacturonase, and xylanase) in the virus-infected and -cured strains (Figs. 5 and S4).

Of the 12 selected genes, four genes encoding manganese peroxidases (*mnp2* and *mnp3*) and laccases (*pox1* and *pox3*), which belong to the group of auxiliary activities (AA) redox enzymes involved in lignin degradation in the CAZy database, exhibited severely down-regulated expression during the vegetative mycelium stage in the POV strain. Among the four AA genes, the most significant difference in expression was observed for *mnp2*, which had 337-fold higher expression in the mycelium of the POVF12 strain than in the POV strain (Fig. 5A–D). Six genes encoding cellulase and β-glucosidase, xylanase, chitinase, polygalacturonase, and amylase, which degrade cellulose, hemicellulose, chitin, pectin, and starch, respectively, and belong to the glycoside hydrolase (GH) group in the CAZy database, showed results similar to those of the lignocellulolytic enzymes in the CAZy AA family. Among the six genes, the most significant difference in expression was observed for a chitinase gene that had 132-fold higher expression in the POVF10 strain than in the POV strain (Fig. 5E–J). In addition, the expression levels of two genes encoding non-CAZymes involved in mushroom growth—lipase and aspartic protease—were noticeably reduced in the mycelium of the POV strain (Fig. 5K,L). All 12 genes encoding CAZymes and non-CAZymes relevant to mushroom growth were affected by PoV infection in the vegetative mycelium of *P. ostreatus*.

Of the 12 genes, the expression levels of genes encoding eight CAZymes and one non-CAZyme also decreased at the mycelium spawn stage in the POV strain in comparison to the two virus-cured strains (Fig. 5). However, the expression patterns of a laccase gene (*pox3*) and a lipase gene were inconsistent between the two virus-cured strains at the mycelium spawn stage. A gene encoding polygalacturonase was not affected by PoV in the spawn stage of *P. ostreatus* (Fig. 5I). Genes encoding extracellular enzymes exhibited substantially down-regulated expression in the mycelium spawn of the POV strain, although the differences were not statistically significant for the four genes encoding POX3, chitinase, amylase, and lipase (Fig. 5D,H,J,K).

Two laccase genes from the CAZy AA family and four genes encoding cellulase, β-glucosidase, chitinase, and amylase from the CAZy GH group exhibited down-regulated expression in the fruiting bodies of the POV strain (Supplementary Fig. S4). Hence, PoV infection affects growth and fruiting body formation in *P. ostreatus* via decreased expression of several genes encoding CAZymes and non-CAZymes.

## Discussion

Most mycoviruses are associated with latent infections of fungal hosts; however, some fungal viruses are reported to impair growth, pigmentation, and virulence of host fungi^1,2,5^. Mycoviruses have been reported to induce various symptoms, including malformation of the fruiting body, in cultivated mushrooms^21^. In this study, the characteristics of PoV infection in *P. ostreatus* were consistent with those of known mycoviral infections in cultivated mushrooms. Therefore, the results of this study will be applicable to investigations of mycovirus–fungal host interactions in other mushroom species. In our previous study, we detected the PoV mycovirus in a malformed fruiting body of a *P. ostreatus* strain and identified an RdRp gene of PoV. All isogenic PoV-cured strains of *P. ostreatus*, in which the virus was removed by the mycelial fragmentation method followed by single colony isolation^17,22^, showed great improvements in growth rate, mycelial dry weight, and pigmentation. Curing the fungus of the virus eliminated infection symptoms^3^. Therefore, many uncharacterized mushroom diseases may be associated with mycoviral infection. However, although the previous study is the first reported comparison of mycelial mass between isogenic PoV-infected and -cured strains of *P. ostreatus*, we could not examine the effects of PoV on fruiting body formation, yield, etc. There is no clear evidence for a cause-and-effect relationship between mycoviral infection and phenotypic alterations in edible cultivated mushrooms such as *P. ostreatus*. Such studies have only been performed for several pathogenic and/or economically important fungi, such as *Cryphonectria parasitica*, *Botrytis cinerea*, and *Sclerotium rolfsii*, to explore the expression of genes potentially related to pathogenic factors in mycovirus–fungal host interactions. *Cryphonectria parasitica*, *B. cinerea*, and *S. rolfsii* infected with CHV1, Bc378V1, and SsHV2, respectively, exhibited significantly reduced growth. Among representative extracellular enzymes, laccase activity is reduced in the mycovirus-infected strains because the laccase gene is one of several regulated genes affected by the presence of viral genes^4,23,24^. However, little is known about other factors associated with fungal–mycoviral interactions, even in industrially important fungi. Therefore, in the present study, we clearly showed that the presence of PoV affected the mycelial branching pattern, fruiting body development, and extracellular enzyme expression in *P. ostreatus*.

The *P. ostreatus* strain infected with PoV in this study exhibited restricted conidial growth and irregular hyphal branching at the colony margin, similar to observations of aggregated mycelia of *Magnaporthe oryzae* infected with MoCV1^25^. These results indicate that colony radial extension in the virus-infected strain was delayed, partly due to the short and agglomerative hyphal branching, which was visible under a microscope, suggesting that PoV may have a decisive effect on organizing and/or regulating the apical elongation process. In several studies of the mechanism of hyphal growth in fungi, fungal hyphae continuously grow and elongate in a specific direction with the aid of lysis of the cell wall at the tip of the hypha^26^. The vesicles of the cell migrate towards the tip of the hypha, where they secrete various enzymes and other compounds during apical elongation. Fungal growth allows for hyphae to be elongated at the hyphal tip depending on lysis of the cell wall^27,28^. For these reasons, it was assumed that mycovirus infection could affect factors involved in the release of various enzymes or other cell wall components. However, we eliminated this possibility because PoV infection had no considerable effect on cell wall integrity according to our experiments using cell wall-disturbing agents. These results suggest that PoV infection may impair the activity of various extracellular enzymes in *P. ostreatus* both directly and indirectly.

According to several reports, the disease symptoms of virus-infected mushrooms include malformed fruiting bodies. Therefore, viral infection is economically damaging due to the deterioration of mushroom quality and yield in species such as *A. bisporus*, *Lentinula edodes*, and *P. ostreatus*^5,15,19,29^. Moreover, the activities of extracellular enzymes that degrade plant biomass are closely associated with improving fruiting body yield^30–32^ because various CAZymes, such as lignocellulolytic enzymes associated with lignocellulose degradation, assist with nutrition supply^18^. Positive activities of extracellular enzymes consequently enhance mycelial growth and fruiting body production; thus, these enzymes are very important for obtaining the highest yield of cultivated mushrooms^20,33^. In this context, PoV infection reduced the total yield, FE, and BE during fruiting body production in *P. ostreatus*. Once again, this result suggests that PoV infection may play a role in hampering the activity of various extracellular enzymes. Moreover, the activity of phenoloxidase, which is involved in lignin biodegradation, decreased substantially in the PoV-infected strain compared to the virus-cured strains. This result confirms that PoV affects extracellular enzyme activity.

CAZy GH enzymes hydrolyze the glycosidic bond between two or more carbohydrates or between a carbohydrate and a non-carbohydrate moiety in basidiomycetes^18^. In this study, because the CAZyme glycoside hydrolases play a critical role in the degradation of wood substrates together with the CAZy AA enzymes, we performed expression analysis of the genes encoding these enzymes to further verify our hypothesis. We clearly showed that the presence of PoV negatively affected the accumulation of mRNA of genes encoding CAZy AA redox enzymes (manganese peroxidase and laccase), CAZy GH enzymes (cellulase, β-glucosidase, xylanase, chitinase, polygalacturonase, and amylase), and non-CAZymes (protease and lipase) in the vegetative mycelium of *P. ostreatus*. Furthermore, these extracellular enzymes were substantially down-regulated in the mycelium spawn due to PoV infection. Two laccase genes from the CAZy AA family and four genes encoding cellulase, β-glucosidase, chitinase, and amylase from the CAZy GH group exhibited down-regulated expression in the fruiting body upon PoV infection. Our results indicate that PoV affects growth and/or fruiting body formation in *P. ostreatus* by decreasing or suppressing the expression of some CAZymes and non-CAZymes. Further studies are necessary to investigate these hypotheses. Edible mushrooms have been used as a resource for enzymes for pharmaceuticals, chemical production, biofuels, food, and consumer products because they are safe and highly nutritious. In particular, effective lignocellulolytic enzymes, which strongly degrade complex lignocellulosic substrates into soluble substances, are produced from cultivated mushrooms^34^. The results of this study suggest that these industrial enzymes should be purified from virus-free commercial mushroom lines.

The factors associated with viral diseases should be further investigated even though substantial data have already been accumulated on the symptoms of infection. Comprehension of the importance of virus-free spawn and the pathogenic mechanisms of viral infection are required for appropriate and efficient production of cultivated mushrooms. Our data can also be applied to investigations of the unexplored relationships between mycoviral infection and phenotypic changes in other mushroom species that are major agricultural products for food, animal feed, enzymes, medicinal compounds, and organopollutant degraders.

## Methods

### Mushroom strains and cultivation

Previously described strains—the PoV-ASI2792 (PoV)-cured and PoV-infected isogenic lines of the edible mushroom *P. ostreatus*—were used for this study^3^. The fungal cultures were maintained on potato dextrose agar (PDA) plates at 25 °C in the dark for 7 days. Sawdust agar plates (5–25% sawdust, 2% rice bran, and 2% agar) were used for the measurement of the radial growth of the colonies under the same culture conditions. Small mycelial cultures were grown on cellophane overlays under the same culture conditions^3^ and collected for the extraction of dsRNA and total RNA. The harvested mycelia were stored at −70 °C for further analyses. PDA plates containing each cell wall-disturbing agent, sodium dodecyl sulfate (SDS) (0.1 and 0.3%) and Congo red (0.005 and 0.01%), were used to assay cell wall integrity of fungal strains at 25 °C in the dark for 5 days. All measurements of radial growth rate on culture plates were performed in at least three biological and three technical replicates. Each inoculum of *P. ostreatus* strains for sawdust-bottle cultivation was grown in potato dextrose broth (PDB) at 25 °C in the dark for 7 days with continuous agitation at 200 rpm.

### Microscopic observation

Fungal mycelia were cultured for 2 days on PDA and viewed under differential interference contrast microscopy (BX53; Olympus, Tokyo, Japan) to analyze the branching pattern of the *P. ostreatus* hyphae. Culture plates containing the fungal hyphae were directly observed at a magnification of 200 × . The ProRes CapturePro software (version 2.8.8; JENOPTIK, Jena, Germany) was used to observe changes in the branching pattern.

### Fruiting body production and yield parameters

For fruiting body cultivation of *P. ostreatus*, sawdust and beet pulp were used as the principal substrates. Sawdust, beet pulp, rice bran, and cotton-seed meal were mixed at a 50:30:10:10 ratio (W/W/W/W) and the mixture was adjusted to a water content of 65% in a polypropylene bottle, as described previously^35^, with slight modifications. Each bottle was inoculated with mycelia grown in PDB and incubated in the dark at 25 °C for 30 days. After the surface of substrate was completely covered with mycelia (M), the sawdust bottles (B) were transferred to horizontal racks in a cropping room at 22–25 °C and 80–85% relative humidity under semidarkness. The mushroom spawn was a pure culture of mycelia growing on a solid substrate. The spawn bottles were then opened, and the mats were rehydrated 2–3 times a day to keep the mycelia moist and induce fruiting body (F) formation^36^. Mycelial spawn and fruiting bodies were stored at −70 °C for further study.

The weights of the harvested fruiting bodies were recorded. The total weight of all fruiting bodies from four flushes was measured and used to calculate total yield and biological efficiency (BE). BE was defined as the ratio of fresh fruiting body weight to substrate dry weight^37,38^. The flush efficiency (FE) was also measured; it was calculated as the ratio of the number of flushed bottles to the total number of bottles^39^.

### Analysis of polyphenol oxidase activity

Polyphenol oxidase activity was measured using the Bavendamm assay^40^. Fungal strains were grown for 5 days on modified Bavendamm’s medium (0.07% aqueous tannic acid, 1.5% malt extract, and 2.0% agar, pH 5.0) in darkness, and assessed by gauging the appearance of a brown oxidation zone around the colonies. The radial growth of the colonies and the diameter of the dark brown zone on Bavendamm’s medium were measured using a calipers (Mitutoyo, Japan). The analyses of polyphenol oxidase activity were performed in three biological and four technical replicates.

### Analysis using qRT-PCR

The dsRNA and total RNA from mycelia, fruiting bodies, and/or sawdust spawn of *P. ostreatus* were extracted as previously described^3,41^. The dsRNA was prepared from the corresponding total nucleic acid preparations. A miniprep method for detection of dsRNA^41^ was applied with slight modifications. The dried mycelia were ground to a fine powder, and the dsRNA was isolated from 200 mg of mycelial powder via CC41 cellulose column chromatography. The dsRNA was dissolved in 40 μl of RNase-free water. Total RNA from fungal samples was purified using TRI Reagent Solution (Ambion, Austin, TX). The quality and quantity of dsRNA and total RNA were evaluated using electrophoresis on 0.8% agarose gels and/or a UV SPECTROstar Nano system (BMG LABTECH, Ortenberg, Germany).

We synthesized cDNA from approximately 0.1–1 μg of dsRNA or total RNA from the mycelia, mature fruiting bodies, and/or sawdust spawn using the GoScript Reverse Transcription system (Promega, Madison, WI). RT-PCR analysis was carried out for the PoV gene encoding the RNA-dependent RNA polymerase (RdRp) protein using the primer pair pov-F and pov-R. The level of viral gene expression in the mycelia and fruiting bodies was performed using the results of qRT-PCR with the PoV-specific primer pair. qRT-PCR was performed in a reaction mixture containing 2 μl of first-strand cDNA and 100 nM each of povq-F and povq-R primers in a total volume of 20 μl. SYBR green-based qRT-PCR was performed using FastStart Essential DNA Green Master mix (Roche, Basel, Switzerland) on a LightCycler 96 system (Roche). Statistical analysis of viral gene expression were calculated *via* the comparative threshold cycle method using the LightCycler 96 qualitative detection module (Roche), as described previously^42^. In addition, relative gene expression levels of target genes in the mycelia, fruiting bodies, and sawdust spawn were performed with the same qRT-PCR method for the viral gene. Detailed primer information is listed in Supplementary Table S1. Expression levels were evaluated in triplicate for each transcript with at least three independent preparations of the same RNA sample. The cytochrome C gene (JGI gene ID: no. 1113744) or actin gene (JGI gene ID: no. 1087906) of *P. ostreatus* was used as the reference gene^30^.

### Statistical analyses

Mycelial colony diameter, fruiting body yield, and area and color intensity of the brown oxidation region were analyzed via one-way analysis of variance using SPSS software (version 18; IBM Corporation, Armonk, NY, USA), with p < 0.05 considered to indicate significant differences between constructs. When a significant F-value was obtained, Duncan’s multiple range test was used to test the significance of the effect (p < 0.05) between virus-cured and virus-infected strains. Relative gene expression levels obtained using qRT-PCR were analyzed using the same statistical test.

## Supplementary information

Supplementary Information.

## Data Availability

All data generated or analyzed during this study are included in this published article (and its Supplementary Information Files).
