# Supplementary material for: The PoV mycovirus affects extracellular enzyme expression and fruiting body yield in the oyster mushroom, Pleurotus ostreatus
Source: Sci Rep. 2020 Jan 23;10:1094. doi: 10.1038/s41598-020-58016-4 (PMC6978373; doi:10.1038/s41598-020-58016-4)
Supplement: Supplementary file 1 — Supplementary Information. [file 41598_2020_58016_MOESM1_ESM.docx]

**Supplementary Information**

**The PoV mycovirus affects extracellular enzyme expression and fruiting body yield in the oyster mushroom, *Pleurotus ostreatus***

Ha-Yeon Song, Nayeon Kim, Dae-Hyuk Kim, and Jung-Mi Kim

**
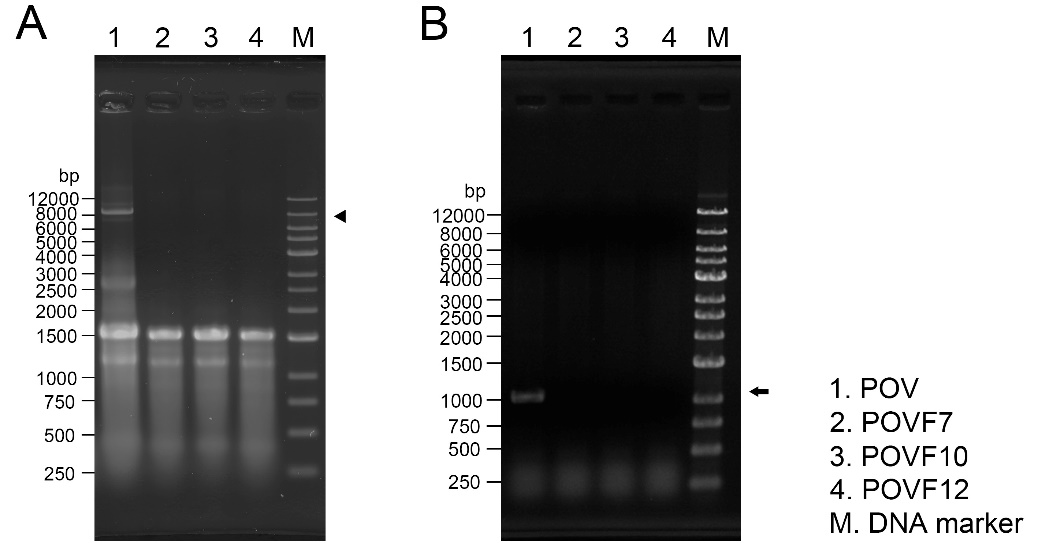
**

**Figure S1. Detection of mycovirus dsRNA in *P. ostreatus* strains.** (A) Ethidium bromide-strained agarose gel showing mycovirus dsRNA in *P. ostreatus* strains. Lane 1 contains dsRNA extracts from the PoV-infected strain (POV) with the characteristic 8-kb viral genome of ASI2792-PoV. Lanes 2, 3, and 4 contain dsRNA extracts from the virus-cured strains POVF7, POVF10 and POVF12, respectively^3^. POVF10 and POVF12 were only used for this study. Lane M contains DNA size marker. The arrowhead indicates the mycovirus genome segment from *P. ostreatus* ASI2792-PoV. (B) Reverse transcription polymerase chain reaction (RT-PCR) analysis of the PoV mycovirus. The dsRNA was extracted and reverse transcribed, and then RT-PCR analysis was performed for the RdRp gene in the PoV mycovirus. Detailed primer information is listed in Supplementary Table S1. Lane numbers correspond to those in (A). The arrow indicates a partial cDNA of PoV from the POV strain.

**
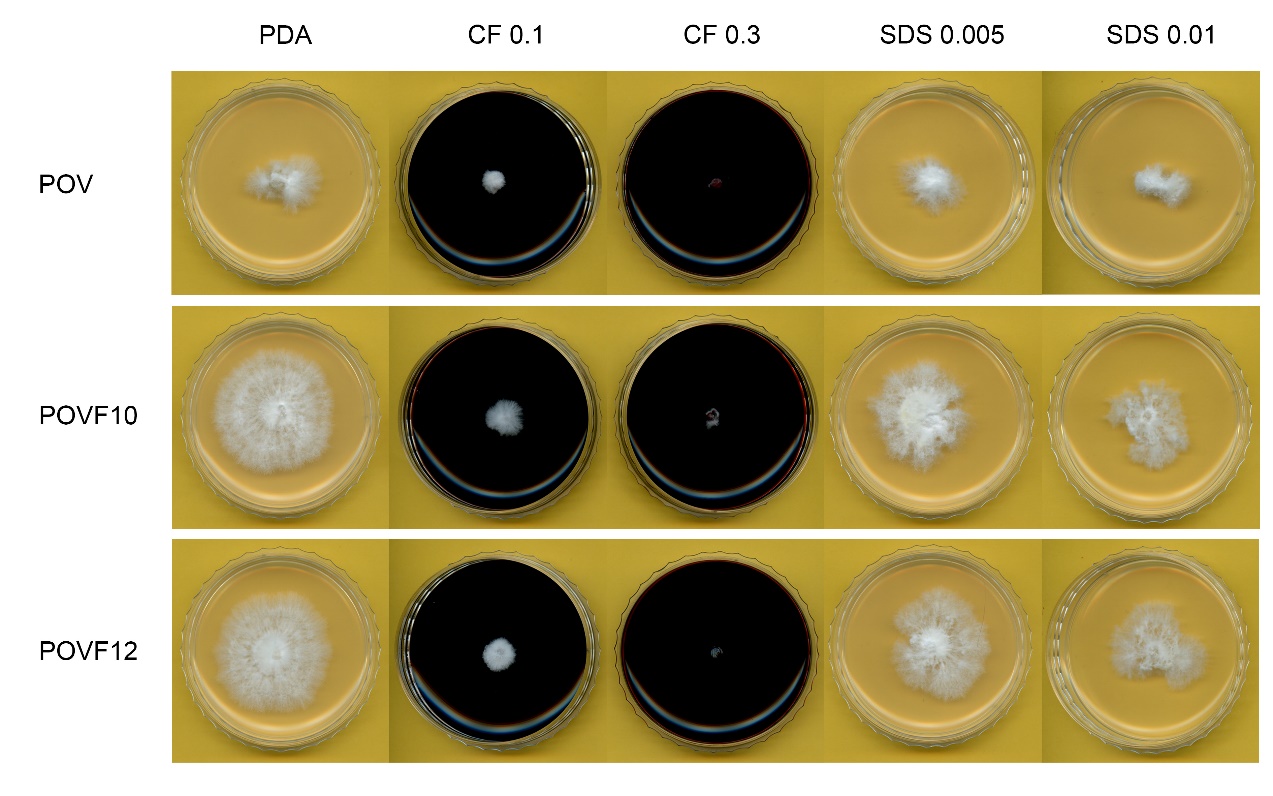
**

**Figure S2. Effect of cell wall-perturbing agents on the fungal growth of a mycovirus-infected *P. ostreatus* strain.** The morphology of colonies grown on potato dextrose agar supplemented with the cell wall-perturbing agents Congo red (CR) (0.1 and 0.3%) and sodium dodecyl sulfate (SDS) (0.005 and 0.01%) after 5 days of cultivation. The strains, indicated on the left, used were a virus-infected strain (POV) and two virus-cured strains (POVF10 and POVF12). Six replicates were used for each strain, and each experiment was performed in duplicate.

**
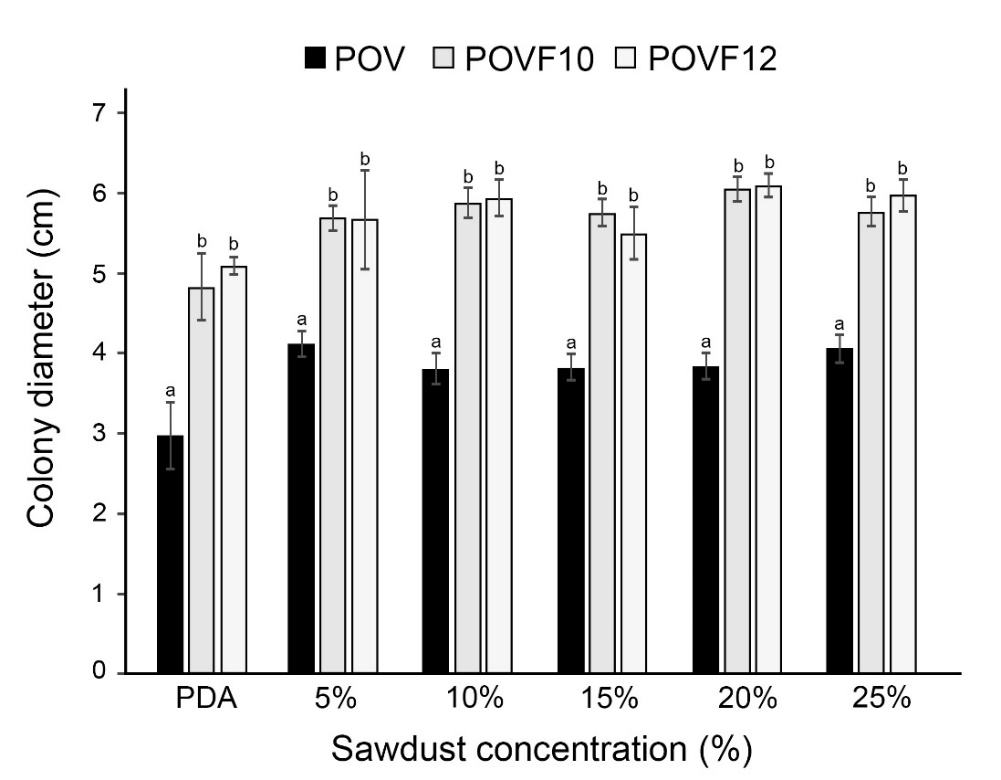
**

**Figure S3. Effect of different concentrations of sawdust on the mycelial growth rate of** **a mycovirus-infected *P. ostreatus* strain.** A virus-infected strain (POV) and two virus-cured strains (POVF10 and POVF12) were grown on sawdust agar medium containing various concentrations of sawdust (5%, 10%, 15%, 20%, and 25%) for 7 days. Data are presented as the means ± standard deviations from five replicates and three independent experiments.

**
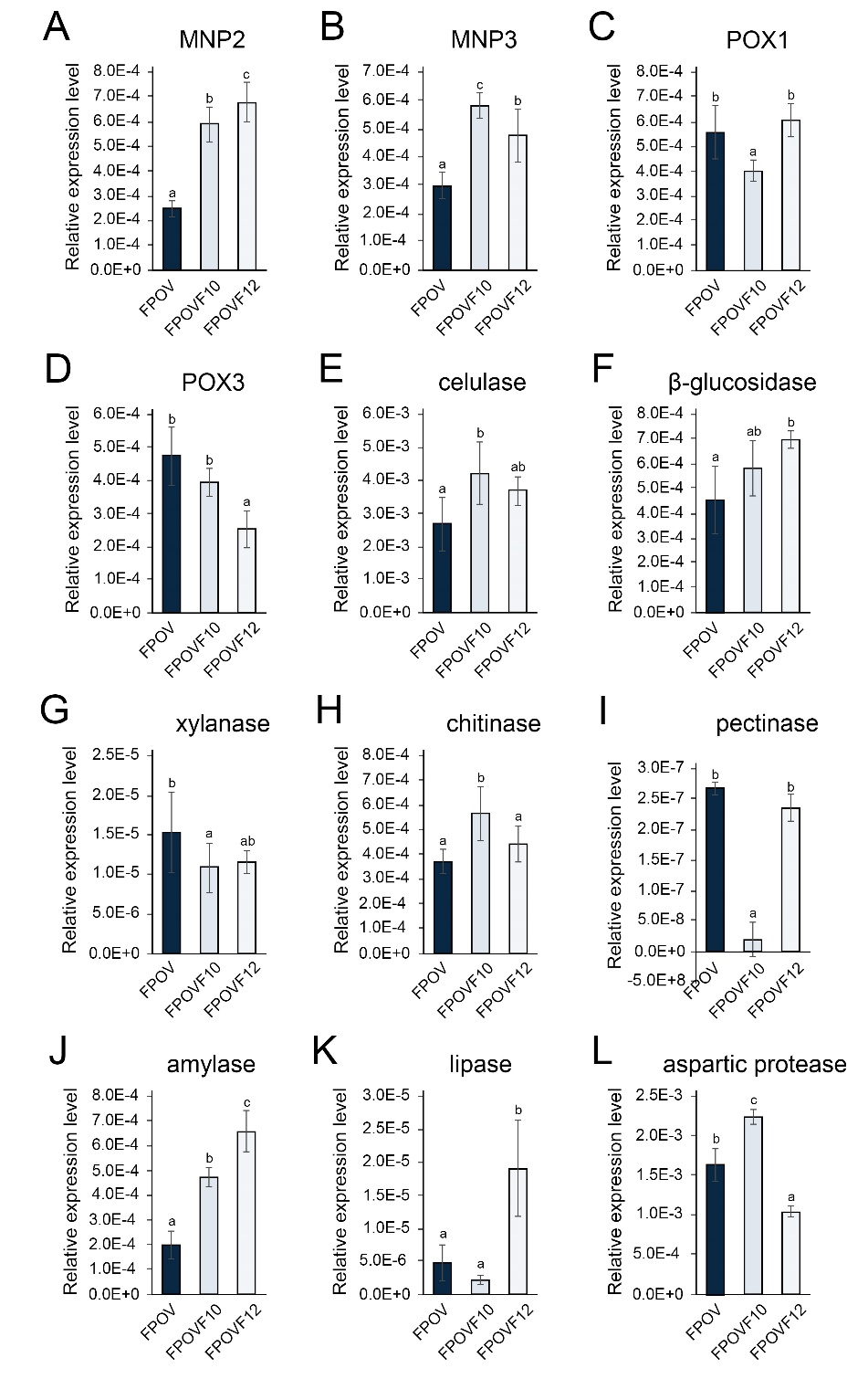
**

**Figure S4. Relative expression levels of the genes coding extracellular enzymes in fruiting bodies of a mycovirus-infected *P. ostreatus* strain.** The expression levels of 12 enzyme-encoding genes in fruiting bodies of a virus-infected strain (POV) were compared to those in the fruiting bodies of virus-cured strains (POVF10 and POVF12). Detailed primer information is listed in Supplementary Table S1. Each error bar represents the standard deviation calculated from at least three independent replicates. Transcript abundances of the 12 genes relative to a housekeeping gene (*cyt-c*) were quantified using qRT-PCR. The dark blue bar indicates analyses of the virus-infected strain (POV). The other two bars indicate analyses of the virus-cured strains (POVF10 and POVF12).

**Table S1. Primers used for quantitative reverse transcription-polymerase chain reaction analyses of specific genes and viral genes in *P. ostreatus*.**

| GenBank  accession no. | Primer name | Sequence (5´ to 3´) |
| --- | --- | --- |
| KX976461  KX976461 | pov-F  pov-R | CCA GCT CAA CCT CGC CTC GTT  GGC GTC AAT TTC TTG GTC GG |
|  | povq-F  povq-R | TCA AGA CCA GAC ATT CCA  ATC CAG GCG ATG TTA GAG |
| KDQ33654 | act-F  act-R | AGT CGG TGC CTT GGT TAT  ATA CCG ACC ATC ACA CCT |
| KDQ24313 | cytc-F  cytc-R | GCC TCA TAA AGT CGG TCC TAA C  CTC AAA TAG GGT GTT CTC GTC C |
| ACM47219 | mnp3-F  mnp3-R | CCT CCT GAC TTT GGC ATC TCA  CAC CTC CTC CAC CTT TGG TT |
| CAB51617 | mnp2-F  mnp2-R | GCC TTT CGA TAG CGT GGA TAA G  GGT CCC TTG CAA CAT TGT CTC |
| BAH90721.1 | pox1-F  pox1-R | CTA TCC TTC GGT ATG CTG GTG  ATA TTG ATG TCT GCG CCT CC |
| KDQ27218.1 | pox3-F  pox3-R | CCC CAT CCT TTC CAT CTT CAC  GTA GTT ATA CAC CGA GCT TCC G |
| KDQ29194 | cel-F  cel-R | GGT AAA GAA GGA GAC AAG  CCG ACA TCA TTA CTA TCA G |
| KDQ33611 | glu-F  glu-R | CAG ATA CCT CTC CGA ACT  GAA CCA GTC ATA ATG TCT CT |
| ABY61039 | xyl-F  xyl-R | GCC AAT ACT CTA TCC AAT G  AAC AAT CCA ACT CCC TAC |
| AFM30903 | chi-F  chi-R | ATC ATA CCT GCC AAT CAT TC  CTG AGC AAC CGT AGT AGT |
| KDQ22750.1 | pec-F  pec-R | CTC CAA CCA AAT AAC ATT CC  GAA GAC ACA ATC CTG TAT CA |
| KDQ32475.1 | amy-F  amy-R | CTA TCA ACC AGT CTC ATA C  CTACAGTAATCACCTTCAC |
| KDQ32910.1 | lip-F  lip-R | CGC TAA GTA GGA TAA TGG  GAC TCA GAA GTA GAG ACT |
| AHA80840.1 | asp-F  asp-R | ATG CGT ATC ATC AGT CTT C  CGT GAT ATG GTT GAC AGA T |

**Table S2. Effect of PoV infection on flush efficiency.**

| Strains | Flushed efficiency (%)^a^ | | | |
| --- | --- | --- | --- | --- |
|  | 1st flush | 2nd flush | 3rd flush | 4rd flush |
| POV | 100.0 | 86.7 | 20.0 | 0.0 |
| POVF10 | 100.0 | 100 | 60.0 | 27.7 |
| POVF12 | 100.0 | 100 | 66.7 | 20.0 |

^a^ Flushed efficiency (%) : the percentage of flushed bottles over the total number of bottles.
